# The emerging roles of N6-methyladenosine (m6A) deregulation in liver carcinogenesis

**DOI:** 10.1186/s12943-020-01172-y

**Published:** 2020-02-28

**Authors:** Mengnuo Chen, Chun-Ming Wong

**Affiliations:** 1 State Key Laboratory of Liver Research, the University of Hong Kong, Hong Kong, China; 2 Department of Pathology, Li Ka Shing Faculty of Medicine, the University of Hong Kong, Hong Kong, China

**Keywords:** Hepatocellular carcinoma, N6-methyladenosine, RNA epigenetics, Epi-transcriptome

## Abstract

Liver cancer is a common cancer worldwide. Although the etiological factors of liver carcinogenesis are well defined, the underlying molecular mechanisms remain largely elusive. Epigenetic deregulations, such as aberrant DNA methylation and histone modifications, play a critical role in liver carcinogenesis. Analogous to DNA and core histone proteins, reversible chemical modifications on mRNA have recently been recognized as important regulatory mechanisms to control gene expression. N6-methyladenosine (m6A) is the most prevalent internal mRNA modification in mammalian cells. m6A modification is important for controlling many cellular and biological processes. Deregulation of m6A modification has been recently implicated in human carcinogenesis, including liver cancer. In this review, we summarize the recent findings on m6A regulation and its biological impacts in normal and cancer cells. We will focus on the deregulation of m6A modification and m6A regulators in liver diseases and liver cancers. We will highlight the clinical relevance of m6A deregulation in liver cancer. We will also discuss the potential of exploiting m6A modification for cancer diagnosis and therapeutics.

## Background

Liver cancer is a common malignancy and lethal disease globally. Although the risk factors for liver carcinogenesis are well defined, the underlying molecular mechanisms remain ambiguous. Liver carcinogenesis is traditionally associated with genetic alterations, including chromosome gain/loss and somatic mutations. Recently, mounting evidence has shown that epigenetic deregulation is also critically involved in liver cancer initiation and progression. Reversible chemical modifications, in particular, methylation, on DNA and core histone proteins are essential for epigenetic control of chromatin structure and gene expression. However, the importance of reversible modifications on RNA has long been underestimated. N6-methyladenosine (m6A) is the most abundant form of internal mRNA modification. RNA m6A modification was first discovered in the 1970s and has gained renewed interest as a new layer of control for gene expression. The recent discovery of m6A methyltransferases and demethylases suggests that m6A modification is a dynamic process. m6A modification plays a crucial role in regulating RNA stability, splicing and translation and has been shown to participate in various biological processes. Deregulation of m6A modification has also been implicated in cancer formation. In this review, we will summarize the recent findings on delineating the functions of m6A modification in normal and cancer cells. We will particularly focus on the impacts of m6A modification on liver carcinogenesis. Finally, we will discuss the recent technological advancements for m6A research, and we will highlight the potential implications of m6A modification in cancer diagnosis and therapeutics.

### Liver cancer

Liver cancer is a common disease and is the fourth most lethal malignancy worldwide. Hepatocellular carcinoma (HCC) is the predominant form of primary liver cancer that accounts for ~ 80% of the cases. In contrast to the decreasing trend of other major cancer types, the incidence of HCC exhibits an increasing trend globally [1]. For instance, HCC incidence in the USA has increased by threefold between 1975 and 2005 [2]. Currently, more than 700,000 new HCC cases are diagnosed annually. HCC shows a specific geographic distribution with higher incidence rates in Eastern Asia and Sub-Saharan Africa [3]. The etiology and risk factors of HCC are relatively well defined. Hepatitis B viral (HBV) infection is the major risk factor for HCC, accounting for 80% of HCC incidence globally. Chronic HBV infection is the most common cause of HCC in China and most of the African counties [4]. Hepatitis C viral (HCV) infection is another prevalent risk factor associated with HCC incidence in Japan and the USA [4]. In Western countries, excessive alcohol consumption with its associated liver cirrhosis is the second most common risk factor for HCC [1]. Recently, nonalcoholic fatty liver disease (NAFLD) has been shown to be another major risk factor for HCC in developed countries. It has been estimated that 10–20% of HCC incidence in the USA is caused by NAFLD [5]. Other risk factors, such as aflatoxin intake and metabolic liver diseases, are also associated with HCC development [1]. The clinical management of HCC remains very challenging. Due to the asymptomatic disease progression and the lack of reliable early diagnostic biomarkers, most HCC patients are diagnosed at the end stage of the disease. Surgical resection is a potential curative treatment but is only applicable in 20–30% of HCC patients, and tumor recurrence is common. Molecularly targeted therapies, sorafenib and lenvatinib, are recommended treatments for unresectable advanced HCC patients, but they can only extend patient survival by 3 months [6]. Nivolumab, an anti-PD1 immune checkpoint therapy, is a new FDA-approved second-line treatment for sorafenib-refractory HCC. It can improve the survival of HCC patients, but only 25% of HCC patients respond to the treatment [7]. Therefore, due to the late diagnosis and limited therapeutic options, HCC remains an incurable disease. Thus, understanding the molecular mechanisms of how HCC develops is essential to advance future diagnostic and therapeutic inventions.

### Reversible chemical modifications on DNA, RNA and histone proteins

#### DNA methylation and histone modifications

Recent whole-genome and whole-exome sequencing analyses have delineated the mutational landscape of HCC and uncovered a number of novel driver mutations [8, 9]. In addition to genetic lesions, accumulating evidence also suggests that epigenetic alterations, in particular, aberrant DNA methylation and histone modifications, are also significantly involved in liver carcinogenesis [10–14]. DNA methylation and histone modifications are reversible and dynamic processes that enable cells to reprogram their transcriptome during cell differentiation and in response to environmental cues. These epigenetic events are collaboratively controlled by a large group of regulatory proteins that can be further subdivided into “writer”, “reader” and “eraser” proteins [15]. DNA methyltransferases, histone acetyltransferases, and lysine methyltransferases are classified as epigenetic “writer” proteins that are responsible for installing the corresponding chemical modifications to the targeted DNA and histone proteins. These chemical modifications can then be recognized by “reader” proteins, such as MBD family proteins for DNA methylation, bromodomain-containing proteins for lysine acetylation and PHD domain-containing proteins for lysine methylation. These “reader” proteins specifically bind to chemically modified DNA or histone proteins and act as scaffolds to recruit other cofactors to modulate chromatin structure and gene expression. Finally, TET family DNA demethylases, histone deacetylases (HDACs), and JMJC family histone demethylases serve as epigenetic “eraser” proteins to remove the existing chemical modifications and enable the reversibility of epigenetic events. Deregulation of epigenetic regulators is frequently reported in human cancers, including HCC. In particular, overexpression of the transcription repressive histone methyltransferases EZH2, SUV39H1, SETDB1 and G9a is implicated in the epigenetic silencing of tumor suppressive genes and microRNAs to promote HCC progression and metastasis [10–14].

#### The emerging field of epitranscriptomics

In addition to DNA and histones, cellular RNAs (mRNA, tRNA, snRNA, etc.) also carry hundreds of distinct post-transcriptional modifications at various sites [16]. Early studies of mRNA modifications focused on the 5′ cap [17]. mRNA 7-methylguanylate (m7G) capping is a highly regulated process essential for the creation of mature mRNA, maintaining mRNA stability, mRNA nuclear exportation and translation initiation [18]. N6-methyladenosine (m6A) has been identified as the most abundant chemical modification on mammalian mRNA and non-coding RNAs and is involved in the regulation of multiple cellular processes [19–22]. After the discovery of m6A, diverse chemical modifications were uncovered on mRNA, including N1-methyladenosine (m1A), N6, 2′-O-dimethyladenosine (m6Am), pseudouridine (Ψ), 5-methylcytosine (m5C), and 5-hydroxymethylcytosine (hm5C). Although these modifications have been known for decades, deciphering their biological roles remains challenging due to the complexity of RNA structure and functions [23, 24]. Interestingly, recent studies have demonstrated that some of these post-transcriptional RNA modifications are reversible and dynamically controlled, indicating that they might have potential regulatory functions similar to those of DNA and histone modifications. In this regard, investigating the landscapes and functions of these reversible RNA modifications is now emerging as a new frontier of research, known as “RNA epigenetics” or “epi-transcriptomics” [25].

### N6-methyladenosine

N6-methyladenosine (m6A) modification refers to the addition of a methyl group at position N6 of adenosine, which is an evolutionarily conserved RNA modification that can be found in most organisms, from bacteria to mammals [26]. m6A modification is identified as the most prevalent chemical modification within eukaryotic mRNA and lncRNA [19–22, 27]. It has been estimated that approximately 0.1 to 0.4% of adenosines in mRNA are subjected to m6A modification, on average, with 2–3 m6A-modified sites per transcript [26, 28, 29].

#### Reversible m6A modification

mRNA modifications were previously considered static, as the half-life of mRNAs is extremely short, leaving limited space for mRNA modification to be functional. Research into mRNA modifications was brought back to the forefront with the discovery of the m6A demethylases FTO [30] and ALKBH5 [31] and the METTL3/METTL14/WTAP m6A methyltransferase complex [32]. These findings are revolutionary since they point out that m6A modification is reversible and can be dynamically regulated, implicating the potential of these proteins in modulating biological processes. Shortly after, with the development of highly specific antibodies and the accessibility of high-throughput sequencing technologies, transcription-wide mapping of m6A sites becomes feasible, which was a milestone in the field of RNA epitranscriptomics [23, 27]. Topology studies into mRNA m6A modification revealed that m6A is enriched in the 3′ UTR, around the stop codon. Approximately 13,000 m6A-modified sites were identified in 5000–7000 genes. Later, studies also revealed the 5′ enrichment of m6A, which is closely linked with protein translation [33, 34]. Overall, m6A modification is more frequently found in ubiquitously expressed genes than in tissue-specific genes, and the latter seems more inclined to be regulated at the transcriptional level. Across human tissues, the global m6A profiles are highly specific in brain tissues and show modest tissue specificity in non-brain tissues. Nevertheless, a subset of tissue-specific m6A sites is sufficient to distinguish different tissue types [35].

#### m6A writer, erasers and readers

**Fig. 1:**
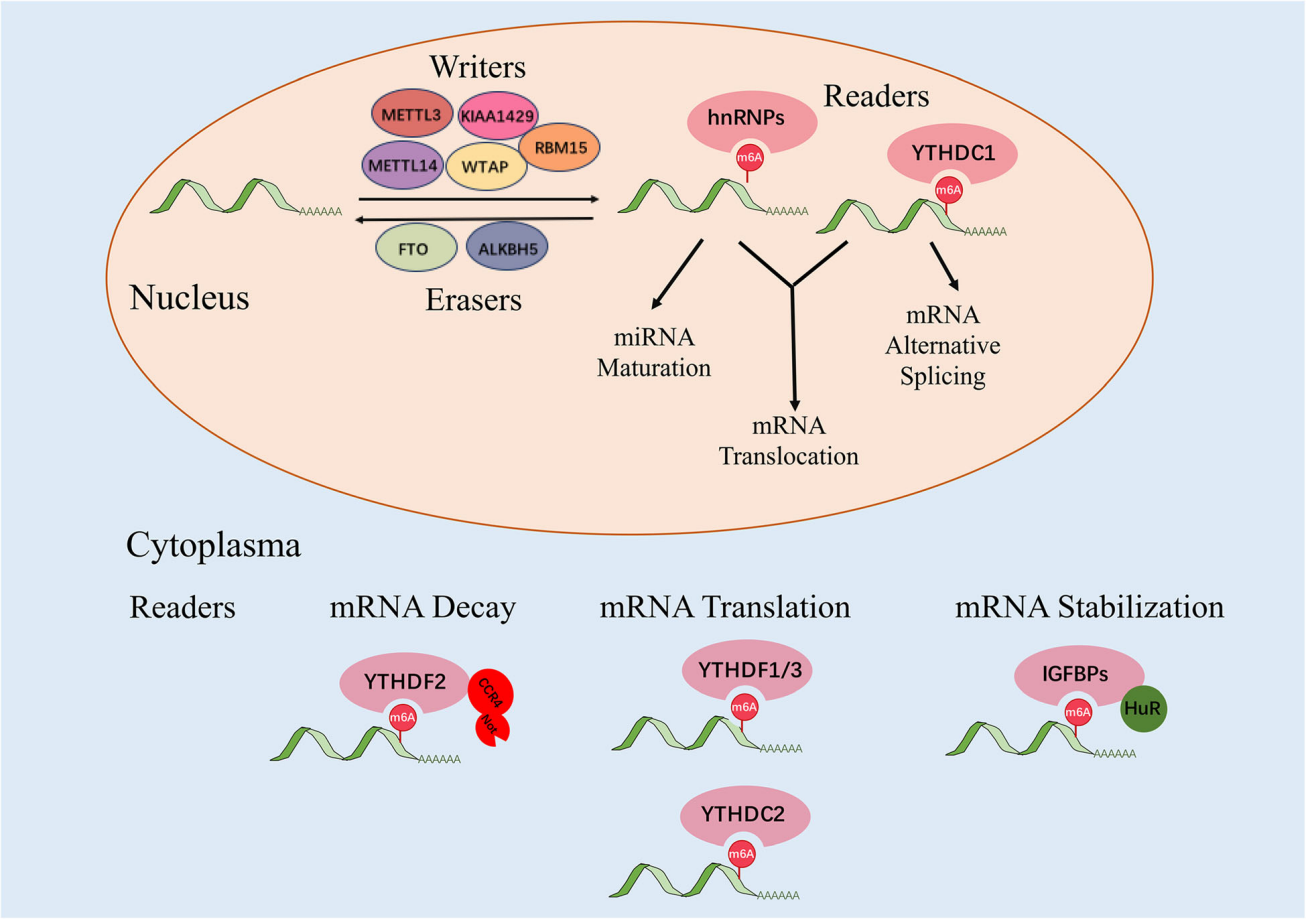
Regulation of m6A modification and its functions in RNA metabolism by m6A “writer”, “eraser” and “reader” proteins

Installation of m6A is a reversible process regulated by the balanced activities of m6A “writer” and “eraser” proteins. The addition of methyl groups to the N6 site of adenine usually occurs within the consensus sequence of RRm6ACH (where R = G or A, and H = A, C or U) [36, 37] and is accomplished by a highly conserved mRNA methyltransferase complex, the so-called m6A “writer” complex. METTL3, METTL14, and WTAP are the core components of this complex [32, 38–41]. Both METTL3 and METTL14 contain a SAM-binding motif. They co-localize in nuclear speckles, form a heterodimer and catalyze the covalent transfer of a methyl group to adenine with the assistance of WTAP [32, 39, 42]. In addition, KIAA1429 and RBM15 have been identified as new components of the m6A “writer” complex [40, 43]. The reversible m6A modification is mediated by m6A “erasers”, FTO and ALKBH5 [30, 31]. Both FTO and ALKBH5 belong to the ALKB family of dioxygenases. While ALKBH5 catalyzes the direct removal of m6A modification, FTO can sequentially oxidize m6A to N6-hydroxymethyladenosine (hm6A) and N6-formyladenosine (f6A), which are moderately stable and can later be hydrolyzed to adenine. The current hypothesis suggests that m6A modification exerts its biological functions either by altering the RNA structure or by recruiting m6A “reader” proteins. There are three classes of m6A “reader” proteins. The class I m6A “reader” proteins contain an evolutionarily conserved YTH (YT521-B homology) domain. This domain folds into a hydrophobic aromatic cage that can directly bind to m6A. The human genome contains five YTH domain proteins, YTHDF1–3 and YTHDC1–2, which are bona fide m6A “readers”. Among these, YTHDF2 was the first identified and is the most studied m6A “reader” protein and influences mRNA stability [34]. YTHDF2 binds to m6A located in the 3′ UTR and localizes the targeted mRNA to processing bodies (P-bodies) for accelerated degradation [34]. Moreover, YTHDF2 also recruits the CCR4-NOT deadenylation machinery to promote mRNA degradation [44]. On the other hand, 5′ UTR m6A has been suggested to enhance mRNA translation efficiency in a cap-independent manner through YTHDF1 [35]. YTHDF1 binding promotes protein translation of m6A-modified mRNA by recruiting the eIF3 translation initiation complex. It has been proposed that the antagonistic functions of YTHDF2 and YTHDF1 may be important in regulating the balance between mRNA decay and translation for their common targets. YTHDC1 is an m6A “reader” mediating RNA splicing. YTHDC1 can recruit the mRNA splicing factors SRSF3 and SRSF10 to promote exon inclusion and exon skipping, respectively. In addition, YTHDC1 also controls the nuclear export of its targets by interacting with SRSF3 and the RNA nuclear exporter NXF1 [45]. Recently, YTHDC2 was found to interact with RNA helicase to positively regulate translation elongation in an m6A-dependent manner [46]. The class II m6A “readers” include three heterogeneous nuclear ribonucleoproteins (hnRNPs), hnRNPC, hnRNPG and hnRNPA2B1. These proteins selectively bind to m6A-containing transcripts though the “m6A-switch”, a mechanism in which m6A weakens Watson-Crick base pairing to destabilize the RNA hairpin structure and thereby exposes the single-stranded hnRNP binding motif. Previous pull-down experiments suggested that hnRNPC and hnRNPG could serve as potential nuclear m6A “readers” to influence mRNA localization and alternative splicing [9]. Another hnRNP member, hnRNPA2B1, binds to m6A-containing primary microRNAs and recruits the microprocessor complex to promote microRNA maturation [47]. IGFBP family proteins, IGFBP1–3, represent the class III m6A “readers”. This class of proteins uses common RNA binding domains, such as the KH domain, to recognize m6A-containing transcripts. However, the exact mechanisms remain unclear. IGFBP proteins preferentially bind to m6A-containing transcripts, and their binding motifs (UGGAC) overlap with the m6A consensus sequence (RRACH). IGFBP proteins exert their functions by recruiting RNA stabilizers, such as HuR, to protect m6A-containing mRNA from degradation. Indeed, the above mentioned m6A “reader” proteins have diversified functions and are involved in regulating almost every step of RNA metabolism, including the stability, translation, and splicing of m6A-containing transcripts (Fig. 1).

#### m6A in physiology and human diseases

mRNA m6A modification has been demonstrated to play important roles in different physiological activities and human diseases. Mounting evidence has shown the importance of m6A methylation in embryonic development and stem cell regulation, including processes such as maintaining pluripotency and promoting differentiation [48–50]. Other functional processes which involve m6A modification, include adipogenesis, development of obesity and pathogenesis of type 2 diabetes [42, 51]. m6A modification has also been implicated in cellular immunological processes. m6A modification facilitates the mRNA degradation of SOCS family genes. SOCSs are negative regulators of the IL-7/STAT pathway, and depletion of SOCSs results in reprogramming of naïve T cells for proliferation and differentiation [52]. A similar mechanism has also been reported for maintaining the immunosuppressive functions of Treg cells, where m6A-mediated suppression of SOCS2 controls the IL-2/STAT5 signaling pathway [53]. In the innate immune response, METTL3-mediated mRNA m6A modification is essential for the translation of the co-stimulatory molecules CD40, CD80 and the TLR4 adaptor TIRAP. Thus, loss of METTL3 impairs dendritic cell maturation and their ability to activate T cells [54].

#### m6A in liver diseases

NAFLD is a risk factor predisposing patients to HCC formation in developed counties and is associated with metabolic syndromes, including obesity and diabetes. Because of the established functions of FTO in obesity and diabetes, it has been proposed that FTO may also play a role in NAFLD development. Several lines of evidence recently supported this hypothesis. FTO is reported to positively regulate adipogenesis. FTO polymorphisms are associated with high BMI and insulin resistance and may contribute to the development of NAFLD. Upregulation of FTO is consistently observed in clinical NAFLD patients as well as in rodent models, suggesting the potential implication of FTO in NAFLD [55, 56].

Apart from regulating eukaryotic mRNAs, m6A modification has also been identified in viral transcripts to affect virus maturation and host response to viral infections [57–59]. HBV/HCV-associated hepatitis is closely linked to liver carcinogenesis. m6A modifications are present in both HBV and HCV. In HBV, m6A modification regulates the half-life of the HBV virus, controls the expression of HBV onco-proteins and regulates the reverse transcriptase of pre-genomic RNAs [60]. In HCV, overexpression of the m6A methyltransferase increases the virus titer, while overexpression of the demethylase decreases the virus titer. Moreover, YTHDF family reader proteins are reported to inhibit HCV replication by competing for binding to Env to prevent virus packaging [61]. Therefore, the deregulation of m6A regulators in host hepatocytes may contribute to the development of viral hepatitis, which is a major risk factor in HCC.

### m6A modification and human carcinogenesis

#### m6A deregulation in human cancers

Emerging evidence suggests that m6A modification is involved in human carcinogenesis. Multiple m6A regulators are reported to be deregulated and function either as oncogenes or tumor suppressors in various cancers. The clinical relevance of aberrant m6A regulator expression has been systematically analyzed in > 10,000 patients across 33 cancer types. It has been found that the overall mutation rates of m6A regulators are low in human cancers. Copy number variants (CNVs) are commonly found in m6A regulators and may have a direct contribution to their expression. Among all, IGFBP family proteins are found to be frequently amplified in different cancer types. On the other hand, FTO and ALKBH5 are prevalently deleted in human cancers. Interestingly, high correlations are found between the expression of different m6A regulators, suggesting extensive crosstalk of the m6A machinery in cancer development [62]. Deregulation of m6A modification and m6A regulators has been implicated to play a role in different cancer functions, including cancer stem cell formation, epithelial–mesenchymal transition (EMT), cancer metabolism, and signaling transduction, by regulating the mRNA stability or protein translation of different downstream targets. In breast cancer, ALKBH5 expression is induced upon hypoxia in a HIF-dependent manner. Overexpression of ALKBH5 reduces m6A modification and stabilizes NANOG mRNA, thereby contributing to breast cancer stem cell formation [63]. m6A modification can control cancer metabolism by modulating autophagy by targeting ATG5/7 and regulating pentose phosphate flux by promoting 6PGD translation [64, 65]. m6A modification also plays an important role in EMT and cancer metastasis by regulating Snail translation in a METTL3- and YTHDF1-dependent manner [66]. In addition, m6A modification also regulates multiple signaling pathways, including the AKT, MYC, NFκB and YAP pathways, to promote cancer growth. It is worth mentioning that the m6A modification landscape and the expression of m6A regulators are highly heterogeneous, implying that the functional implications of m6A modification may vary across different cancer contexts. In acute myeloid leukemia (AML), m6A modification plays an essential role in leukemia cell survival and proliferation by regulating various mRNA metabolic activities. AML has the highest expression of METTL3 and METTL14 among all cancer types. METTL3 and METTL14 function as oncogenes in AML. Loss of METTL3 or METTL14 induces cell cycle arrest and apoptosis in leukemia cells [67]. Paradoxically, overexpression of the m6A demethylase FTO is also found in AMLs carrying gene translocations of FTL3-ITD, MLL-AF9 or PML-RARA. In this context, FTO is reported to serve as an oncogene in leukemogenesis, in which FTO targets ASB2/RARA to promote AML cell growth and inhibit ATRA-induced differentiation [68]. In the context of glioblastoma (GBM), the m6A demethylases FTO and ALKBH5 have also been reported to act as oncogenes. However, unlike AML, METTL3 and METTL14 serve as tumor suppressors to inhibit GBM stem cell self-renewal and tumor progression [69, 70]. Further investigations are required to delineate the enigmatic roles of m6A modification and m6A regulators in different cancer types. Nevertheless, the above evidence converges to support that, similar to DNA methylation and histone modifications, RNA epigenetic alteration is also a common event in human cancers.

#### The implications of m6A modification in liver carcinogenesis

The importance of m6A modification in liver carcinogenesis has been increasingly recognized in recent years. Growing efforts have begun to demystify the complicated roles of m6A modification and the deregulation of m6A regulators in HCC. By comprehensively analyzing the expression of the m6A “writers” and “erases” in TCGA and Hong Kong HCC cohorts, Chen et al. reported that METTL3 was significantly upregulated in human HCC compared to non-tumorous liver controls. Consistently, the global m6A modification level is also elevated in human HCC. METLL3 possesses oncogenic functions in human HCC, and knockdown of METTL3 attenuates HCC tumorigenicity and lung metastasis in an orthotopic liver xenograft model. Mechanistically, METTL3 promotes m6A modification on the 3′ end of the mRNA of the tumor suppressor gene SOCS2, which therefore promotes the degradation of SOCS2 mRNA through a YTHDF2-dependent mechanism. This study provided the first proof-of-concept model to demonstrate METTL3-mediated m6A hypermethylation as a new mechanism for epigenetic silencing of tumor suppressor gene expression in human cancers [71]. Interestingly, apart from HCC, the METTL3/m6A/SOCS axis has also been found to be conserved in T cells and iPSCs to regulate T cell homeostasis and pluripotency, respectively [52, 72]. In another study, METTL3 was reported to be critical for EMT in HCC. Li et al. found that the global mRNA m6A level was significantly increased during EMT. Loss of METTL3 impaired invasion, metastasis and EMT in HCC both in vivo and in vitro. The authors further identified Snail, an important transcription factor involved in EMT, as the target of METTL3-mediated m6A modification. METTL3 works collaboratively with YTHDF1 to promote the protein translation of Snail. These findings explain how overexpression of METTL3 contributes to HCC metastasis. In fact, high expression of METTL3, YTHDF1 and Snail is correlated with poor prognosis in HCC patients [66]. Similarly, WTAP and KIAA1429, another two components of the m6A “writer” complex, are also upregulated in HCC and correlated with poor patient survival [73, 74]. In contrast, Ma et al. reported that METTL14 expression was decreased in human HCC and was associated with tumor recurrence. The authors also reported that METTL14 interacted with the microprocessor protein DGC8 to promote the maturation of miR-126. Downregulation of METT14 attenuated miR-126 expression and thereby promoted HCC metastasis [75]. In summary, different components of the m6A “writer” complex have been reported to play either oncogenic or tumor suppressive roles during HCC progression, but the majority of these findings support the oncogenic role of METTL3 in human HCC. The diversity of roles between METTL14 and other m6A “writers” is apparently controversial. The reasons for the above conflicting findings remain an open question but might reflect the heterogeneity of HCC cell lines and clinical samples. Further investigations are required to settle these contradictory findings and clarify the roles of different components of the m6A “writer” complex in liver carcinogenesis.

Differential expression of m6A “erasers” has also been found in primary liver cancers. Overexpression of FTO is observed in HCC tissues, which indicates a poor prognosis. The knockdown of FTO induces cell cycle arrest and suppresses the colony formation ability of HCC cells, which is accompanied by an increase in the global m6A level. FTO stimulates the demethylation of PKM2 mRNA and facilitates its protein translation to promote HCC progression [76]. However, the downregulation of FTO at the protein level is found in intrahepatic cholangiocarcinoma (ICC), the second most common form of primary liver cancer. Loss of FTO in ICC correlates with cancer aggressiveness and poor prognosis. Functionally, knockdown of FTO reduces the apoptosis of ICC cells and confers resistance to cisplatin treatment. In contrast, ectopic expression of FTO reduces ICC cell anchorage-independent growth and metastasis [77]. These conflicting functions of FTO in the two major types of primary liver cancer again raise the possibility of context-specific m6A landscapes and functions between HCC and ICC.

Like “writers” and “erasers”, multiple m6A “readers” have also been implicated in liver cancer. Hou et al. reported that YTHDF2 expression was downregulated in human HCC, which was correlated with more aggressive clinicopathological features. Functionally, in both human and mouse HCC, loss of YTHDF2 disrupts the m6A-dependent mRNA decay of IL11 and SERPINE2 mRNA. Overexpression of IL11 and SERPINE2 reshapes the HCC microenvironment by promoting inflammation and vascular remodeling. Interestingly, hypoxia has been found to be responsible for the negative regulation of YTHDF2 expression. Treatment with PT2385, a HIF-2a inhibitor, rescues YTHDF2 expression in HCC [78]. Of note, the expression change of YTHDF2 in HCC is also controversial. Yang et al. identified miR-145 as a post-transcriptional regulator of YTHDF2. miR-145 binds to the 3′ UTR of YTHDF2 mRNA, which significantly suppresses its expression. Interestingly, miR-145 is frequently downregulated in HCC and negatively correlates with YTHDF2 expression, implying that YTHDF2 is likely upregulated in this HCC cohort [79]. IGF2BPs have been identified as new readers of mRNA m6A modification. Functionally. IGF2BPs play a positive role in supporting HCC growth in an m6A-dependent manner. The knockdown of IGF2BPs in HepG2 cells reduces mRNA stability and causes suppression of MYC and other target gene expression at the post-transcriptional level [80]. In addition, IGFBP1 also promotes SRF expression in Huh-7 cells by impairing microRNA-mediated post-transcriptional regulation in an m6A-dependent manner.

**Fig. 2:**
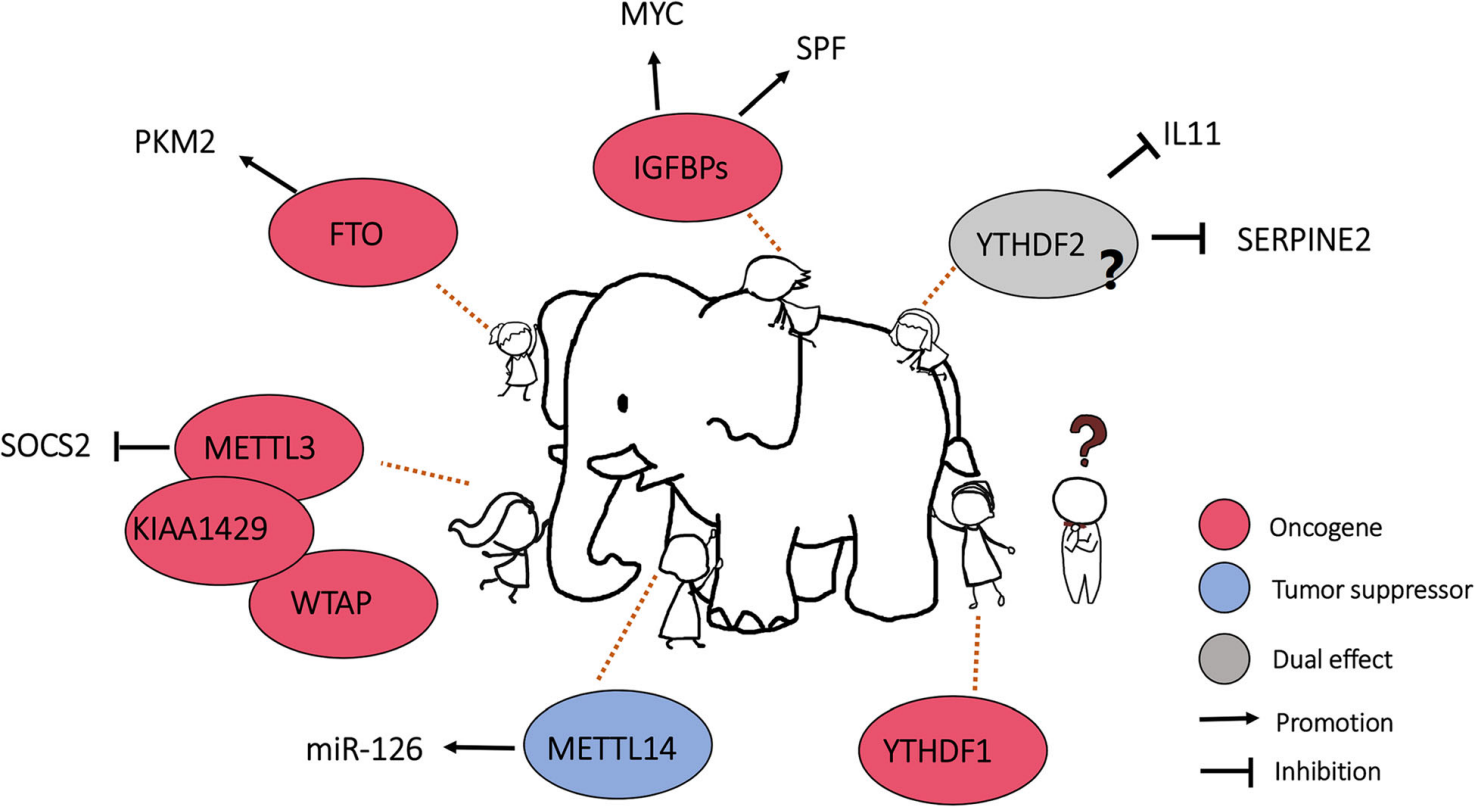
Deregulation of m6A modification and m6A regulators in human HCC

As a new frontier of epigenetic research, mRNA m6A modification has gained increasing attention, and its involvement in different biological processes and disease models has been recently reported. Since epigenetic alterations are frequently observed in human cancers, plenty of evidence in the recent few years uncovering the important regulatory functions mediated by m6A modification is not surprising. The RNA epigenetic studies in human HCC have encountered a major problem in that some of the studies above have reported contradictory results on the expression patterns or functions of different m6A regulators. All the discrepant findings of the above studies underscore the complexity of m6A modification and its regulatory enzymes in human HCCs. It is likely that each of the above studies only reveals a part of the whole picture, akin to the parable of “the blind men and the elephant” (Fig. 2). Further investigations will be required to reconcile these seemingly contradictory findings to generate a unified model.

### Future prospects

#### New m6A profiling technologies

m6A detection and quantification can be achieved by high-speed liquid chromatography after labeling with radioactive [methyl-H^3^] methionine or LC-MS/MS with deuterium-labeled AdoMet [32, 81]. These methods allow the detection and comparison of the overall m6A level with high sensitivity. However, sequence-specific information is lost during RNase digestion; therefore, the above methods are not suitable for studying m6A modification at specific adenosine residues. SELECT, a single-base elongation and ligation-based qPCR amplification method, has been developed for measuring m6A levels at specific adenosine residues [82]. SELECT is a flexible and convenient approach and is expected to facilitate the detailed characterization of site-specific m6A modifications in the future. Beyond site-specific studies, many groups have also developed various high-throughput assays to delineate the m6A modification profiles on a transcriptome-wide scale. Methylated RNA immunoprecipitation sequencing (MeRIP-Seq or m6A-seq) is the mainstay method for transcriptome-wide m6A profiling. This technique, analogous to ChIP-Seq in the mapping of histone modifications, relies on a specific anti-m6A antibody to pull down m6A-containing RNA fragments, which can then be mapped by next generation sequencing (NGS). Through this approach, more than 10,000 putative m6A modification sites have been identified in the human transcriptome, more commonly found in the 3′ UTR, adjacent to the stop codon and within long exons [23]. However, this technique detects m6A-containing RNA fragments rather than specific m6A-modified sites. The resolution of this method is therefore limited by the size of the RNA fragment pulled down, typically 100–200 nt. The resolution of m6A profiling can be improved by combining antibody-based immunoprecipitation with the photo-crossing-linking method, as is seen with PA-m6A-Seq (photo-cross-linking-assisted m6A sequencing) and miCLIP (m6A individual nucleoside resolution and cross-linking immunoprecipitation). By detecting the mutations generated by crosslinking the anti-m6A antibody with neighboring nucleotides during immunoprecipitation, these methods can achieve high or even single-nucleotide resolution m6A mapping [83]. miCLIP is currently the most widely used technique for transcriptome-wide m6A mapping. However, the above transcriptome-wide methods are highly dependent on the antibody. Therefore, the anti-m6A antibody used inevitably affects their sensitivity and specificity. In fact, it is known that the current anti-m6A antibodies used cannot distinguish m6A and m6Am modifications, which may complicate data interpretation [83]. To circumvent the limitation of antibody bias, some antibody-independent methods have been recently developed. m6A-REF-Seq (m6A-sensitive RNA-endoribonuclease-facilitated sequencing) uses the methylation-sensitive RNA endoribonuclease MazF to discriminate m6A and unmodified adenosine. MazF specifically cleaves RNA at the ACA motif, which can be blocked by the presence of m6A modification. In NGS analysis, MazF digestion results in sequencing reads sharply terminating at the unmethylated ACA site, while the presence of m6A modification protects the RNA from digestion and allows the sequencing reads to extend beyond the ACA motif. This method is not only convenient but also quantitative, as the ratio of sequencing read splits at the ACA motif (i.e., unmethylated sites) versus sequencing reads with internal ACA sequences (i.e., m6A-modified sites) can be calculated [83, 84]. Nevertheless, the ACA sequence only accounts for 16% of the canonical RRACH motifs, and MAFz digestion cannot cover the majority of putative m6A sites. Discovery of new m6A-sensitive endoribonucleases that recognize different motifs may help to expand the application of this technique. DART-Seq in another antibody-independent method for m6A mapping. DART-Seq uses an APOBEC1-YTH fusion protein to recognize m6A-modified residues and induce a C to U mutation at adjacent sites that can be readily detected by NGS [85]. More excitingly, the recent development of third-generation single-molecule sequencing technology empowers direct detection of nucleotide sequence and modifications in RNA, which is emerging as an ideal platform for transcriptome-wide m6A profiling. In this approach, a single-stranded RNA is propelled through a protein nanopore in a flow cell. When passing through the nanopore, different nucleotides generate a change in the ionic current flow, and these electrical signals can be used to determine the RNA sequence. In addition to different nucleotides, the presence of different RNA modifications can also result in a detectable current change that provides an unprecedented opportunity to study the comprehensive RNA modification landscape of full-length RNA transcripts [86]. Nevertheless, deconvolution of the complicated electrical signal to identify RNA sequences and modifications remains challenging due to the limitations of computational algorithms. Most recently, Lorenz et al. demonstrated the ability of nanopore-based sequencing to detect m6A modification in endogenous mRNA transcripts. This quickly evolving m6A detection method is expected to greatly accelerate the discovery and validation of m6A modification sites in the human transcriptome. This information will generate a more comprehensive picture of the m6A landscape in human cancers and eventually may facilitate the development of new biomarkers for cancer diagnosis and molecular classifications.

#### m6A RNA editing technology

m6A-seq delineated the current global m6A modification profiles and identified a large number of m6A modification sites in the human transcriptome. However, the biological implications of site-specific m6A modifications remain largely unexplored. With the advancement of CRISPR technology, different m6A editing systems have recently been developed, which may substantially accelerate m6A research in the near future. In the CRISPR/Cas9 m6A editing system, a fusion protein of the catalytic domains of METTL3 and METT14 (M3-M14) is tagged to the N terminus of an RNA-targeting dCas9 mutant. This dCas9-M3-M14 complex can be directed to specific RNA sequences by a sgRNA and a PAM antisense oligo (PAMer). This engineered m6A “writer” complex has demonstrated the ability of site-specific m6A modification. This system is a very powerful tool to study the functional impact of site-specific m6A modifications. Using this system, Liu et al. showed that inducing m6A modification at the 5′ UTR of Hsp70 promoted protein translation. However, the installation of m6A modification on the 3′ UTR of ACTB mRNA resulted in RNA degradation. On the other hand, the RNA-targeting dCas9 can also be fused with the m6A demethylases FTO or ALKBH5 to erase site-specific m6A modification. It has been shown that removal of the m6A modification in lncRNA MALAT1 at A2577 resulted in structural change and altered the interaction with the RNA binding protein hnRNPC [87]. A similar dCas9-FTO system has also been reported by another group [88]. In another study, Rauch et al. made use of the newly identified RNA-guide RNA targeting CRISPR/Cas13 system to interrogate the functional consequence of the binding of different m6A “reader” proteins to the targeted RNA. In this system, catalytically inactive dCas13b was fused with the N-terminal part of YTHDF1 or YTHDF2 without the m6A-binding domain. The engineered dCas13b-YTHDF1 and dCas13b-YTHDF2 proteins could be directed to specific RNA targets by the complementary sequence on gRNAs independent of the m6A modification status of the targeted RNA. These fusion proteins retained the reported function of YTHDF1 and YTHDF2. When tethered to the firefly luciferase mRNA, dCas13b-YTHDF1 slightly reduced the mRNA stability but significantly activated its protein translation. However, the binding of dCas13b-YTHDF2 resulted in the depletion of the firefly luciferase reporter at both the mRNA and protein levels. Furthermore, recruitment of the dCas13b-YTHDF2 protein also promoted the degradation of endogenously expressed putative m6A-modified mRNAs, including KRAS and PPIB mRNAs, in HEK293 cells [89].

#### Diagnosis and therapeutic potential

Deregulation of m6A “writer”, “eraser” and “reader” proteins in different types of human cancers has been recently reported. Some of these deregulations are associated with increased cancer aggressiveness and poor patient survival. In human HCC, overexpression of METTL3 and YTHDF1 was associated with poor survival of HCC patients [66, 71]. Therefore, the expression of m6A regulators may be a potential biomarker for molecular classification and prognostic prediction in HCC patients. A recent study demonstrated that m6A levels could be detected in circulating tumor cells (CTCs) by LC-ESI/MS/MS. In a small cohort of lung cancer patients, the authors reported that the m6A level was significantly elevated in CTCs compared to whole blood samples. This study demonstrates that the detection of m6A levels in CTCs might be a potential non-invasive approach for cancer diagnosis [90]. Further investigations should confirm whether the deregulation of m6A and m6A regulators is an early event in human carcinogenesis that can be detected in premalignant lesions, which is important to evaluate the potential of utilizing m6A and m6A regulators for early cancer diagnosis.

Deregulation of epigenetic regulators has been linked to the development of drug resistance. METTL3 is overexpressed in pancreatic cancer and promotes cancer cell resistance to gemcitabine, 5-fluorouracil, cisplatin and irradiation [91]. In glioma, overexpression of METTL3 is involved in glioma stem-like cell maintenance and radioresistance [92]. In cervical cancer, the upregulation of FTO enhanced chemo-radiotherapy resistance by activating β-catenin and excision repair pathways [93]. FTO is also upregulated in multiple tyrosine kinase inhibitor (TKI)-resistant leukemia cells, resulting in demethylation and overexpression of a subset of survival genes. Knockdown of FTO remarkably sensitizes resistant leukemia cells to TKI treatments. Importantly, combined treatment with an FTO inhibitor and nilotinib works synergistically to overcome the TKI resistance phenotype and suppress leukemia growth in both in vitro and in vivo models [94]. These studies highlight the therapeutic value of targeting m6A regulators in drug-resistant tumors.

Immune checkpoint therapy is emerging as a new direction for cancer treatment. By targeting PD1 in cytotoxic T cells or PD-L1 in cancer cells, immune checkpoint therapies activate the adaptive immune system to eliminate cancer cells. Yang et al. showed that knockdown of FTO sensitizes melanoma cells to interferon gamma and anti-PD1 treatments [95]. m6A modification is also implicated in the neoantigen-specific T cell immune response. Han et al. found that the growth of ovalbumin (OVA)-expressing B16 melanoma cells was remarkably attenuated in immunocompetent YTHDF1-deficient mice when compared to the wild-type control. YTHFD1 deficiency resulted in an increase in CD8+ T cell and NK cell infiltration and a reduction in the MDSC population in the tumor. Depletion of CD8+ T cells significantly abolished the tumor-suppressive phenotypes of YTHDF1-deficient mice. Mechanistically, knockout of YTHDF1 deaccelerates the protein translation of m6A-modified mRNAs of lysosomal cathepsins in dendritic cells, which results in a delay of degradation of ingested neoantigens and thereby facilitates antigen cross-presentation and T cell cross-priming by dendritic cells. Importantly, the knockout of YHTDF1 substantially sensitizes the anti-tumor response of anti-PD-L1 treatment. The above findings suggest that targeting m6A and m6A regulators could be a potential therapeutic strategy to improve the outcomes of immune checkpoint therapy [96].

There is an increasing need to develop potent and specific inhibitors for m6A regulatory proteins. Rhein, a natural product, is the first identified FTO inhibitor and competes with m6A-containing RNA for binding to the catalytic domain of FTO [97]. However, rhein is not an FTO-specific inhibitor, and it has been reported that rhein can also inhibit other ALKB family demethylases [98]. Meclofenamic acid (MA) is another FTO inhibitor and shows high selectivity in inhibiting FTO over ALKBH5 [99]. More recently, based on the structural-guide design approach, the MA derivatives FB23 and FB23B were developed as new FTO inhibitors. Treatment with FB23–2 significantly deaccelerated AML proliferation and suppressed the progression of AML in PDTX mouse models [100]. In another recent study, by computer-aided virtual screening of 1323 FDA approved drugs, Peng et al. identified entacapone, a catechol-O-methyltransferase inhibitor originally used for the treatment of Parkinson’s disease, as a new FTO inhibitor. Entacapone inhibits FTO by competitively binding with both m6A-modified RNA substrates and the co-factor α-KG. Treatment with entacapone increases m6A levels in human cell lines and reduces body weight and blood glucose levels in diet-induced obese mice in an FTO-dependent manner [101]. Because entacapone is an FDA-approved drug and has a safe toxicity profile, it could be readily repurposed for the treatment of other FTO-related diseases, including cancers. Unfortunately, to date, there are no specific inhibitors for m6A regulatory proteins other than FTO. Further structural studies and large-scale chemical screening are required to develop specific inhibitors for targeting deregulated m6A regulatory proteins. New specific inhibitors will not only enhance the mechanistic understanding to dissect the functional impactions of m6A and m6A regulatory proteins in human carcinogenesis but also provide new therapeutic opportunities for cancer patients.

## Conclusions

RNA m6A modification is emerging as a new layer of post-transcriptional regulation of gene expression. The implications of m6A modification in human carcinogenesis have been demonstrated in different cancer types, including HCC. Deregulation of m6A regulators modulates the expression of different downstream targets by mediating mRNA stability and translation efficiency. However, further studies are required to address the heterogeneity and complexity of m6A modification and m6A regulators in HCC development. The recent development of m6A mapping approaches and m6A editing tools will greatly facilitate m6A studies at a single-nucleotide level, which may advance this exciting field. Future effectors are also required to identify cancer-specific m6A modifications for early diagnosis and develop specific inhibitors to target m6A regulators for therapeutic purposes.

## Data Availability

Not Applicable.

## Abbreviations

ALKBH5: AlkB Homolog 5 RNA demethylase
AML: Acute myeloid leukemia
CRISPR: Clustered regularly interspaced short palindromic repeats
CTCs: Circulating tumor cells
EMT: Epithelial–mesenchymal transition
FTO: Fat Mass and Obesity-associated
GBM: Glioblastoma
HBV: Hepatitis B virus
HCC: Hepatocellular Carcinoma
HCV: Hepatitis C virus
hnRNP: Heterogeneous Nuclear Ribonucleoproteins
ICC: Intrahepatic cholangiocarcinoma
IGFBP1–3: Insulin Like Growth Factor Binding Protein 1–3
m6A: N6-methyladenosine
MA: Meclofenamic acid
METTL14: Methyltransferase Like 14
METTL3: Methyltransferase Like 3
NAFLD: Nonalcoholic fatty liver disease
NGS: Next generation sequencing
SOCS: Suppressor Of Cytokine Signaling
TCGA: The Cancer Genome Atlas
TKI: Tyrosine kinase inhibitor
WTAP: WT1 Associated Protein
YTH: YT521-B homology
YTHDC1–2: YTH Domain Containing 1–2
YTHDF1–3: YTH N6-Methyladenosine RNA Binding Protein 1–3
